# Single-step autoantibody profiling in antiphospholipid syndrome using a multi-line dot assay

**DOI:** 10.1186/ar3421

**Published:** 2011-07-21

**Authors:** Karl Egerer, Dirk Roggenbuck, Thomas Büttner, Barbara Lehmann, Annushka Kohn, Philipp von Landenberg, Rico Hiemann, Eugen Feist, Gerd-Rüdiger Burmester, Thomas Dörner

**Affiliations:** 1 Department of Rheumatology and Clinical Immunology, Charité-Universitätsmedizin Berlin, Charitéplatz 1, 10117 Berlin, Augustenburger Platz 01, 13555 Berlin, Germany; 2 GA Generic Assays GmbH, Ludwig-Erhard-Ring 3, 15827 Dahlewitz/Berlin, Germany; 3 Institut für Labormedizin, Bürgerspital Solothurn, Schöngrünstrasse 42, 4500 Solothurn, Switzerland; 4 Lausitz University of Applied Sciences, Großenhainer Straße 57, 01968 Senftenberg, Germany

## Abstract

**Introduction:**

Diagnosis of antiphospholipid syndrome (APS) still remains a laboratory challenge due to the great diversity of antiphospholipid antibodies (aPL) and their significance regarding APS-diagnostic criteria.

**Methods:**

A multi-line dot assay (MLDA) employing phosphatidylserine (PS), phosphatidylinositol (PI), cardiolipin (CL), and beta2-glycoprotein I (β2 GPI) was used to detect aPL, immunoglobulin G (IgG) and immunoglobulin M (IgM) in 85 APS patients, 65 disease controls, and 79 blood donors. For comparison, anti-CL and anti-β2 GPI IgG and IgM were detected by enzyme-linked immunosorbent assay (ELISA).

**Results:**

The level of agreement of both methods was good for anti-CL IgG, moderate for anti-CL IgM, very good for anti-β2 GPI IgG, and moderate for anti-β2 GPI IgM (kappa = 0.641, 0.507, 0.803 and 0.506, respectively). The frequency of observed discrepancies for anti-CL IgG (1.75%), anti-CL IgM (3.93%), anti-β2 GPI IgG (1.75%), and anti-β2 GPI IgM (0.87%) was low (McNemar test, *P *< 0.05, not-significant, respectively). Sensitivity, specificity, positive (+LR) and negative (-LR) likelihood ratios for at least one positive aPL antibody assessed by ELISA were 58.8%, 95.8%, 14.1, and 0.4, respectively, and for at least three positive aPl IgM and/or one positive aPL IgG by MLDA were 67.1%, 96.5%, 19.3, and 0.3, respectively. The frequency of IgM to PI, PS and CL, and combination of three or more aPL IgM detected by MLDA was significantly higher in APS patients with cerebral transient ischemia (*P *< 0.05, respectively).

**Conclusions:**

The novel MLDA is a readily available, single-step, sensitive diagnostic tool for the multiplex detection of aPL antibodies in APS and a potential alternative for single aPL antibody testing by ELISA.

## Introduction

Antiphospholipid syndrome is an autoimmune clinical entity comprising as core manifestations venous or arterial thrombosis and recurrent fetal loss [1-3]. The APS can occur primary in isolation or secondary in association with other autoimmune conditions, notably systemic lupus erythematosus (SLE). The most life threatening manifestation of APS is called catastrophic APS characterized by multi-organ failure due to occlusion of small blood vessels [4]. According to a recently updated international consensus statement, the association of at least one clinical criterion with one laboratory criterion determines the diagnosis of APS. Persistent elevation of aPL antibodies and/or lupus anticoagulant over 12 weeks constitutes the diagnostic criterion [5]. The generic term aPL antibodies comprises antibodies that interact with phospholipids directly and particularly those that target cofactor proteins binding to such phospholipids. Antiphospholipid antibodies that interfere with phospholipid-dependent steps in the coagulation cascade constitute the lupus anticoagulant (LAC) determined by functional clotting tests. Antiphospholipid antibodies reacting with pure phospholipids alone appear to belong to the natural antibody repertoire and may be elevated during certain infections [6,7]. In fact, such aPL antibodies to CL, PI, phosphatidylcholine and PS have been demonstrated in APS patients and appear to be relevant for the laboratory diagnosis of APS. However, aPL antibodies recognizing cofactor proteins in complex with phospholipids have been reported to have a closer association with clinical manifestations in APS [8-13].

Consequently, aPL antibodies have been shown to be a rather heterogeneous group with distinct associations with clinical symptoms of APS. Therefore, despite the revised APS consensus criteria, diagnosis of APS remains challenging [14]. According to the updated consensus statement, anti-β2 GPI and anti-CL IgG and IgM antibodies and LAC are recommended for aPL antibody testing [5]. In case of seronegativity of these aPL antibodies and clinical signs consistent with APS, further aPL should be assessed requiring laboratory flexibility and appropriate tests. With regard to the detection techniques applied, antiphospholipid antibodies have been mainly detected by solid-phase ELISA so far.

Thus, state-of-the art laboratory diagnosis of APS requires running several ELISA simultaneously in routine laboratories, which generates substantial costs. There is clearly a need for multiplex tests detecting aPL antibodies. Multi-line dot assays or other multiplex techniques like biosensor analysis may overcome this shortcoming by providing the opportunity to detect several aPL antibodies simultaneously as reported for multiplex assessment of autoantibodies in other autoimmune diseases like SLE [15,16].

In this study, we demonstrated the practicability of a unique multi-line dot technique for simultaneous determination of aPL antibodies against four different targets. The objective of the study was to investigate the hypothesis whether this assay technique would be an alternative for aPL antibody testing in the serological diagnosis of APS. By providing reliable results, this new approach would be cost-effective and time-saving compared to single detection of aPL antibodies.

## Methods

### Patients and controls

Eighty-five patients with APS (71 females, 14 males, median age 45 years, range 16 to 77 years) were included in this study. Diagnosis of APS had been established by characteristic clinical and serological criteria according to the international consensus criteria [5]. Eight (9.4%) of the 85 patients with APS suffered from adverse outcomes in pregnancy, whereas 62 (72.9%) had a history of arterial and/or venous thrombosis. Fifty-seven (67.1%) of the latter suffered from deep venous thrombosis (DVT). Eighteen (21.2%) of the patients suffering from APS met the diagnostic criteria for SLE. Thirteen (15.3%) APS patients demonstrated cerebral transient ischemic attack (TIA) (10/85) and/or ischemic stroke (5/85). Two APS patients each with thrombotic events suffered from Sjögren's syndrome and scleroderma, respectively. One APS patient with thrombotic events suffered from rheumatoid arthritis and two patients from spondyloarthropathies.

As a disease control group of patients suffering from diseases unrelated to APS (DC), sera of 65 patients with clinical symptoms suspicious of APS but without positive laboratory tests were included (60 females, 5 males; median age: 46 years, range 19 to 76 years). Ten (15.4%) of those 65 patients suffered from recurrent abortions unrelated to APS and 17 (26.2%) demonstrated DVT of non-APS causes. Five (7.7%) APS patients demonstrated TIA and one patient had an ischemic stroke. Four patients from this group were diagnosed with undifferentiated connective tissue disease, three patients met the diagnostic criteria for SLE, four patients for Sjögren's syndrome, and one patient for scleroderma. Complete physical examinations had been performed in all patients with APS and controls by one investigator (TD).

As controls, 79 sera from normal healthy subjects (NHS), anonymous age- and sex matched donors were used.

The study was approved by the local ethical committee (EA1/001/06). Written informed consent was obtained from each patient. All sera had been stored at -20°C.

### Monoclonal anti-phospholipid antibodies

For comparison of ELISA and MLDA detection of aPL, the human monoclonal antibody (mAb) EY2C9 (IgM) reacting against epitopes on β_2_GPI was obtained from the Center of Disease Control. In the presence of β2 GPI, EY2C9 bound to CL coated plates.

Furthermore, the human monoclonal IgG antibodies HL5B and HL7G, generated from a patient with primary APS and recurrent cerebral microemboli, were included [17,18]. Both antibodies are of the IgG2 subtype with lambda light chains; however, they differ in their aPL reactivity.

### Assessment of lupus anticoagulant by phospholipid-dependent coagulation test

Lupus anticoagulant (LAC) was detected according to the guidelines of the International Society on Thrombosis and Haemostasis [19].

### ELISA for the detection of antibodies to cardiolipin and β2 GPI

Antibodies to CL and β2 GPI in the patient sera were detected using commercially available solid-phase ELISA employing purified CL and β2 GPI in complex with cardiolipin as solid-phase antigens, respectively (GA Generic Assays GmbH, Dahlewitz, Germany). Assessment of aPL antibodies was conducted according to the instructions of the manufacturer. Sera were considered positive when their concentration exceeded the cut-off of 10 GPU or MPU for IgG and IgM respectively.

### Multi-line dot assay for the detection of aPL antibodies

Antibodies to CL, β2 GPI, PS, and PI in the patient sera were detected using a commercially available MLDA employing purified human β2 GPI (The Binding Site, Birmingham, UK) and phospholipids according to the recommendations of the manufacturer (GA Generic Assays GmbH, Dahlewitz, Germany). Briefly, the phospholipids CL, PS, and PI (Sigma, Taufkirchen, Germany) and the protein β2 GPI were sprayed onto (polyvinylidene difluoride) PVDF membrane in lines for immobilization as described for glycolipids recently [20] Processed strips were read out densitometrically employing a scanner with the evaluation software Dot Blot Analyzer (Additional file, Figure S1, GA Generic Assays GmbH, Dahlewitz, Germany).

### Statistical analysis and determination of assay performance characteristics

Intra- and inter-assay coefficients of variations (CV) were calculated. The functional assay sensitivity for aPL detection was determined as described previously [21]. Differences between groups were tested by chi-square test and Fisher's exact test with two-tailed probability as appropriate. Inter-rater agreement statistics was applied for within group comparison. *P-*values < 0.05 were considered significant. Assay performance including sensitivity, specificity, positive and negative likelihood ratio and receiver-operating characteristics (ROC) curve analysis were determined using Medcalc statistical software (Medcalc, Mariakerke, Belgium).

## Results

### Characterisation of the multi-line dot assay

Assay performance analysis of ELISA and MDLA demonstrated similar data regarding assay variation and functional assay sensitivity. Data are shown in Additional file 1. For comparison of ELISA and MLDA techniques, the human mAb IgG HL7G and HL5B, and the human IgM EY2C9 were run in anti-CL and anti-β2 GPI ELISAs and in the novel MLDA. The latter mAb obtained from the Center of Disease Control reacted readily in both anti-CL and anti-β2 GPI IgM ELISAs and demonstrated a strong IgM reactivity to β2 GPI in the MLDA. The level of aPL specific IgM required to reach the cut-offs in the respective ELISAs were similar to the concentration of specific IgM revealing a cut-off band in MLDA. In contrast, the aPL IgG mAb HL7G and HL5B demonstrated reactivity in MLDA only.

### Profiling of aPL antibodies by ELISA and MLDA

Furthermore, sera from patients with APS (*n *= 85), DC (*n *= 65), and NHS as controls (*n *= 79) were assessed for comparison of aPL antibody in ELISA and the novel MLDA (Table 1).

**Table 1:** Number of aPL antibody positive sera investigating 85 APS patients, 65 DC patients, and 79 NHS in ELISA and in the MLDA

	ELISA					MLDA									ELISA or MDLA
	**anti-CL**		**anti-β2 GPI**		**anti-CL or anti-β2 GPI**	**anti-PS**		**anti-PI**		**anti-CL**		**anti-β2 GPI**		**anti-PS, anti-PI, anti-CL or anti-β2 GPI**	
	**IgG**	**IgM**	**IgG**	**IgM**		**IgG**	**IgM**	**IgG**	**IgM**	**IgG**	**IgM**	**IgG**	**IgM**		

APS *n *= 85	45	32	27	33	50	28	17	7	3	39	33	31	27	57	63
DC *n *= 65	1	1	0	0	1	1	0	0	0	2	3	0	3	7	8
NHS *n *= 79	1	0*	0	4	5	1	1	0	0	2	6*	0	5	8	12

* *P *< 0.05 for the comparison of ELISA and MLDA

APS, antiphospholipid syndrome; anti-β2 GPI, anti-beta2-glycoprotein I; anti-CL, anti-cardiolipin; anti-PI, anti-phosphatidylinositol; anti-PS, anti-phosphatidylserine; DC, disease controls; ELISA, enzyme-linked immunosorbent immunoassay; MLDA, multi-line dot assay; NHS, normal healthy subjects.

Patients suffering from APS demonstrated a significantly higher frequency of anti-CL IgG (*P *< 0.000001, respectively), anti-CL IgM (ELISA: *P *< 0.000001, respectively; MLDA: *P *< 0.000001, *P *= 0.0002, respectively), anti-β2 GPI IgG (*P *< 0.000001, respectively) and anti-β2 GPI IgM (ELISA: *P *< 0.000001, respectively; MLDA: *P *< 0.000001, *P *= 0.000049, respectively) compared to DC patients and NHS in both ELISA and MLDA. Anti-PS IgG and IgM detected by MLDA occurred significantly more frequently in patients with APS compared to the control groups (*P *< 0.000001, respectively and *P *= 0.000001, *P *= 0.000077, respectively). The number of anti-PI IgG positive patients was significantly elevated in the APS group in contrast to DC patients and NHS (*P *= 0.004573, *P *= 0.014091, respectively), whereas anti-PI IgM did not demonstrate a significant higher prevalence in this patient cohort in comparison with the control groups.

Remarkably, all anti-PI positive samples found in the APS patient cohort also showed a positive reactivity with the respective anti-PS isotype antibody. Furthermore, all anti-PS positive samples showed a positive anti-CL isotype antibody response, too. Regarding aPL IgG positive APS patients, all seven anti-PI IgG positive patients demonstrated positive anti-PS, anti-CL, and anti-β2 GPI IgG by MDLA either (*P *= 0.000447). Twenty (95.2%) of the remaining 21 anti-PS IgG positive APS patients revealed a similar pattern of positive anti-CL and anti-β2 GPI IgG by MDLA, while only one patient showed anti-CL IgG only (*P *< 0.000001). The two anti-PS IgG positive individuals in the control groups demonstrated anti-CL IgG only.

Comparing ELISA and MLDA data, there was no statistical difference in the frequencies of positive anti-CL and anti-β2 GPI IgG antbodies detected by either method (Table 2). The agreement between both methods was assessed as good for anti-CL IgG (kappa = 0.641, 95% confidence interval (CI): 0.541 to 0.767), moderate for anti-CL-IgM (kappa = 0.507, 95% CI: 0.357 to 0.657), very good for anti-β2 GPI IgG (kappa = 0.803, 95% CI: 0.685 to 0.921) and moderate for anti-β2 GPI IgM (kappa = 0.506, 95% CI: 0.352 to 0.659) according to inter-rater agreement statistics.

**Table 2:** Comparison of anti-CL and anti-β2 GPI antibodies detected in ELISA and MLDA

anti-CL IgG		MLDA			anti-CL IgM		MLDA		
		**positive**	**negative**	**n**			**positive**	**negative**	**n**
ELISA	positive	32	15	47	ELISA	positive	22	11	33
	negative	11	171	182		negative	20	176	196
	n	43	186	229		n	42	187	229
**anti-β2 GPI IgG**		**MLDA**			**anti-β2 GPI IgM**		**MLDA**		

		positive	negative	n			positive	negative	n
ELISA	positive	24	3	27	ELISA	positive	21	16	37
	negative	7	195	202		negative	14	178	192
	n	31	198	229		n	35	194	229

Investigating 85 APS patients, 65 DC patients, and 79 NHS in ELISA and MDLA, no statistical difference could be detected for both techniques. According to the McNemar test, differences for anti-CL IgG (1.75%, 95% CI: -2.97% to 6.05%), anti-CL IgM (3.93%, 95% CI: -1.25% to 8.33%), anti-β2 GPI IgG (1.75%, 95 CI: -1.33% to 3.78%), and anti- β2 GPI IgM (0.87%, 95% CI: -4.11% to 5.67%) were not significant (*P *= 0.5563, *P *= 0.1508, *P *= 0.3438, and *P *= 0.1508, respectively). anti-β2 GPI, anti-beta2-glycoprotein I;

anti-CL, anti-cardiolipin; ELISA, enzyme-linked immunosorbent immunoassay; MLDA, multi-line dot assay.

It should be noted that comparing LAC with combined MDLA data in the group of patients with APS, there was no statistical difference according to McNemar's test (difference: 5.88%; 95% CI: -11.26% to 22.29%; *P *= 0.5677). In terms of MLDA, patient samples were scored positive when IgG or IgM antibodies to PI, PS, CL, or β2 GPI were detected above the cut-off for bands. Lupus anticoagulant and MLDA data were significantly related (contingency coefficient = 0.324, *P *= 0.0016). Strength of agreement between both methods was fair (kappa = 0.303, 95% CI: 0.152 to 0.455).

Patients suffering from APS showed significantly more multiple positive samples detected by ELISA (41/85) and MLDA (44/85) compared with DC patients (ELISA: 0/65, MLDA: 2/65, *P *< 0.000001, respectively) and NHS (ELISA: 0/79, MLDA: 6/79, *P *< 0.000001, respectively) (Additional file 1, Table S1). The frequency of multiple positive samples detected in the APS patient cohort by ELISA was not significantly different from the frequency obtained by MLDA (*P *= 0.759115). Interestingly, the MLDA detected a higher number of samples (39/85) with three and more positive aPL in the APS patient group compared to ELISA (29/85); however, this difference was not significant.

By comparing both detection methods in the DC group, there was also no significant difference in the frequency of multiple positive samples (*P *= 0.496124). In the NHS group; however, only the number of samples demonstrating three or more positive aPL antibodies was not significantly different (*P *= 1.0). There was only one NHS demonstrating three aPL IgM antibodies (anti- β2 GPI, anti-CL, and anti-PS) simultaneously in the MLDA.

### Assay performance of aPL detection by ELISA and MLDA

The assay performance characteristics for the respective aPL detected by ELISA are summarized in Table 3. The specificities for all four detected aPL antibodies were remarkably high ranging from 97.2% (anti- β2 GPI IgM) to 100.0% (anti- β2 GPI IgG). The most sensitive aPL antibody assessed by ELISA was anti-CL IgG revealing a sensitivity of 52.9%. Taking into account the laboratory criteria for APS requiring at least one positive aPL antibody, combined ELISA data showed a sensitivity of 58.8% with a specificity of 95.8%. This resulted in a +LR of 14.1 and a -LR of 0.4.

**Table 3:** Performance characteristics of ELISA for IgG and IgM to CL and β_2_GPI

	Sensitivity	95% CI	specificity	95% CI	+LR	95% CI	-LR	95% CI
**anti-CL IgG**	52.9	41.8 to 63.9	98.6	95.1 to 99.8	38.1	9.5 to 153.2	0.5	0.4 to 0.6
**anti-CL IgM**	37.6	27.4 to 48.8	99.3	96.2 to 100.0	54.2	7.5 to 309.6	0.6	0.5 to 0.7
**anti-β2 GPI IgG**	31.8	22.1 to 42.2	100.0	97.5 to 100.0	∞		0.7	0.6 to 0.8
**anti-β2 GPI IgM**	38.8	28.4 to 50.0	97.2	93.0 to 99.2	14.0	5.1 to 38.1	0.6	0.5 to 0.8

Investigating 85 APS patients, 65 DC patients, and 79 NHS, sensitivity, specificity, and likelihood ratios were calculated using a cut-off of 10 U/ml for all ELISA

anti-β2 GPI, anti-beta2-glycoprotein I; anti-CL, anti-cardiolipin; +LR, positive likelihood ratio; -LR, negative likelihood ratio.

The MLDA performance characteristics are given in Table 4. Specificities for the respective aPL antibodies ranged from 93.8% (anti-CL IgM) to 100.0% (anti-PI IgG and IgM, anti- β2 GPI IgG). The most sensitive aPL antibody assessed by MLDA was also anti-CL IgG revealing a sensitivity of 45.9%. In contrast to the ELISA performance, the combined assessment of aPL by the MLDA revealed a remarkable sensitivity of 67.9% with a specificity of 89.6% (Table 5). This resulted in a low +LR of 6.5 and an acceptable -LR of 0.4. The moderate specificity of the MLDA was in particular due to a significantly higher number of false positive anti-CL IgM determined in the DC and NHS (9/144) groups compared to the ELISA data (1/144, *P *= 0.019742).

**Table 4:** Performance characteristics of MLDA for IgG and IgM to PS, PI, CL, and β_2_GPI

	Sensitivity	95% CI	Specificity	95% CI	+LR	95% CI	-LR	95% CI
**anti-PS IgG**	32.9	23.1 to 44.0	98.6	95.1 to 99.8	23.7	5.8 to 97.1	0.7	0.6 to 0.8
**anti-PS IgM**	20.0	12.1 to 30.1	99.3	96.2 to 100.0	28.8	3.9 to 212.6	0.8	0.7 to 0.9
**anti-PI IgG**	8.2	3.4 to 16.2	100.0	97.5 to 100.0	∞		0.9	0.9 to 1.0
**anti-PI IgM**	3.5	0.7 to 10.0	100.0	97.5 to 100.0	∞		1.0	0.9 to 1.0
**anti-CL IgG**	45.9	35.0 to 57.0	97.2	93.0 to 99.3	16.5	6.1 to 44.6	0.6	0.5 to 0.7
**anti-CL IgM**	38.8	28.4 to 50.0	93.8	88.5 to 97.1	6.2	3.1 to 12.3	0.6	0.6 to 0.8
**anti-β2 GPI IgG**	36.5	26.7 to 47.6	100.0	97.5 to 100.0	∞		0.6	0.5 to 0.8
**anti-β2 GPI IgM**	31.8	22.1 to 42.8	94.4	89.4 to 97.6	5.7	2.7 to 12.1	0.7	0.6 to 0.8

Investigating 85 APS patients, 65 DC patients, and 79 NHS, sensitivity, specificity, and likelihood ratios were calculated.

anti-β2 GPI, anti-beta2-glycoprotein I; anti-CL, anti-cardiolipin; +LR, positive likelihood ratio; -LR, negative likelihood ratio.

**Table 5:** Comparison of the performance characteristics of ELISA and MLDAf

	Sensitivity	95% CI	specificity	95% CI	+LR	95% CI	-LR	95% CI
**at least one aPL antibody by ELISA**	58.8	47.6 to 69.4	95.8	91.1 to 98.5	14.1	6.3 to 31.5	0.4	0.3 to 0.6
**at least one aPL antibody by MLDA**	67.9	56.8 to 77.6	89.6	83.4 to 94.0	6.5	4.0 to 10.8	0.4	0.3 to 0.5
**at least one aPL IgG or at least three aPL IgM by MLDA**	67.1	56.0 to 76.9	96.5	92.1 to 98.9	19.3	8.1 to 46.3	0.3	0.2 to 0.5

Sensitivity, specificity and likelihood ratios were calculated using a cut-off of 10 U/ml for all ELISA investigating 85 APS patients, 65 DC patients, and 79 NHS.

anti-β2 GPI, anti-beta2-glycoprotein I; anti-CL, anti-cardiolipin; aPL, anti-phospholipid antibody; ELISA, enzyme-linked immunosorbent immunoassay; MLDA, multi-line dot assay; +LR, positive likelihood ratio; -LR, negative likelihood ratio.

### Optimal assay performance of MLDA regarding APS diagnosis

False positive aPL IgM findings in control patients and NHS in particular were the main cause of a lower specificity compared with ELISA data. Consequently, by increasing the cut-off for the number of positive aPL IgM to three and considering each positive aPL IgG antibody, the resulting specificity of 96.5% for the MLDA increased to a similar value obtained in ELISA (Table 5). The remarkable sensitivity of 67.1% for this approach was only slightly lower compared to the sensitivity considering each single aPL antibody in the MLDA. This sensitivity, requiring at least three positive aPL IgM antibodies and/or at least one positive aPL IgG, however, is still higher compared to the value obtained by ELISA (58.8%) considering at least one positive aPL antibody. The corresponding +LR for the MLDA demonstrated a remarkable increase to 19.3 with a -LR of 0.3 presenting the optimal assay performance regarding the diagnosis of APS for all approaches in this study cohort.

### Clinical association of MLDA findings

Regarding the number of patients with clinical symptoms and additional disease in the cohort of APS patients, 57/85 patients suffered from DVT and 18/85 patients fulfilled the diagnostic criteria of SLE, which was significantly higher in comparison with the DC group (17/65, *P *< 0.000001; 3/65, *P *= 0.003918; respectively). In contrast, the number of patients suffering from TIA and/or ischemic stroke (13/85) and recurrent miscarriages (8/85) did not differ significantly to the DC group (6/65, 10/65, respectively).

Assessing all aPL antibodies detected by ELISA and MLDA, only anti-PI IgM (3/10), anti-PS IgM (5/10), and anti-CL IgM (7/10) antibodies detected by the MLDA demonstrated a significant higher prevalence in the APS patients suffering from TIAs compared with the remaining APS patients (0/75, *P *= 0.001215; 12/75, *P *= 0.024165; 26/75, *P *= 0.041781; respectively). Interestingly, the detection of three or more aPL IgM antibodies by MLDA also revealed a significant higher prevalence in this APS patient cohort (5/10 vs 11/75; *P *= 0.018102). More detailed analyses of the 13 APS patients with ischemic stroke and/or TIA also revealed a significant higher prevalence of anti-PI IgM (3/13) and anti-CL IgM (9/13) antibodies compared with the remaining APS patients (0/72, *P *= 0.002826; 24/72, *P *= 0.027352; respectively). Again, the detection of three or more aPL IgM antibodies by MLDA demonstrated a significant higher prevalence in this APS patient group (6/13 vs 10/72, *P *= 0.01376).

Remarkably, the absence of anti-CL IgM in the eight APS patients with pregnancy morbidity assessed by ELISA was significantly different to its occurrence in the remaining 77 APS patients (*P *= 0.022343). Only one patient of this group demonstrated positive anti-CL IgM assessed by MLDA alone and one patient anti- β2 GPI IgM detected by ELISA together with several aPL IgG by both methods. All other aPL IgM antibodies assessed by the MLDA were negative, whereby the absence of anti-β2 GPI IgM detected by this technique almost reached statistical significance (*P *= 0.051114).

The prevalence of LAC was significantly higher in the 18 patients with APS and SLE compared to the remaining 67 APS patients without SLE (18/18 vs 44/67, *P *= 0.002159), which was not seen for any other aPL antibody investigated.

## Discussion

According to the classification criteria for APS, the laboratory testing of aPL antibodies play an essential role in diagnosing APS in comparison with other diagnostic criteria for autoimmune disorders and subsequently lead to important treatment decisions [5,22]. In this context, the clinical diagnosis of APS has to be confirmed by and depends on appropriate laboratory diagnosis. Therefore, screening for aPL antibodies is usually not recommended to avoid false-positive laboratory diagnosis as with other lab investigations [14].

In fact, assessment of aPL still remains a diagnostic challenge regarding both immunological tests for direct aPL antibody detection and indirect assessment, thereof, by coagulation tests.

Apart from technical aspects and assay characteristic performance, cost-effectiveness, and availability of robust tests are required. Multiplex detection of aPL antibodies by MLDA may address these issues and provide a reliable tool for aPL antibody profiling suggested for other autoimmune diseases like rheumatoid arthritis [15,23]. The use of aPL antibody profiles according to the number and type of positive aPL antibody is recommended and may identify patients at higher risk, although standardization of assays remains a challenge [5,24,25].

To the best of our knowledge, this study reports the first MLDA for the detection of multiple aPL IgG and IgM antibodies. Results obtained by this assay technique were in good agreement with data obtained by ELISA and demonstrated no statistical difference regarding the laboratory diagnosis of APS. Enzyme-linked immunosorbent assays have been the main method used for the detection of aPL antibodies due to obvious immunochemical peculiarities. For example, microtiter plates activated by gamma radiation provide the opportunity to detect disease-specific anti-β2 GPI antibodies in the absence of phospholipids [26,27]. However, dot blot assays have also been used to determine disease-specific anti-β2 GPI antibodies [28,29]. The PVDF membrane employed in the MLDA investigated in this study appears to induce the same conformational changes in the β2 GPI polypeptide since no significant different frequencies of both anti- β2 GPI IgG and IgM was found in APS patients comparing both assay techniques. Interestingly, the human mAb aPL IgG antibodies demonstrated an even better reactivity in the MLDA compared to ELISA.

It should be noted that this study provides data consistent with the conclusion that not all clinically relevant aPL are only directed against proteins [6,30,31]. The phospholipids employed in the MLDA for the detection of aPL antibodies are immobilized in the absence of co-factors like β2 GPI and the only source thereof could be proteins from the patient samples analyzed. Despite the significantly higher rate of false-positive anti-CL IgM detected by MLDA in NHS compared with ELISA, all other aPL frequencies assessed by MLDA in the respective cohorts were not significantly different from ELISA data. In this context, the IgM detection systems compared to the IgG tests may be influenced by some naturally occurring antibodies, in particular among sera from the NHS group. By increasing the cut-off for positivity to at least three positive aPL IgM and/or one aPL IgG assay performance of MLDA for the serological diagnosis of APS demonstrated even better data than ELISA. The sensitivity for APS reached a remarkable value of 67.1% with a corresponding +LR of 19.3 which is similar to values reported elsewhere [32].

Interestingly, apart from LAC for SLE, only aPL IgM detected by MLDA revealed a significant association with clinical symptoms in the 85 patients with APS. The remarkable significantly higher prevalence of IgM to PI, PS, and CL in APS patients suffering from TIA and of IgM to PI and CL in APS patients with TIA and/or ischemic stroke warrants further investigation. Several reviews covered neurological symptoms in patients with APS [33,34]. The association of aPL to pure phospholipids has been shown in patients suffering from multiple sclerosis to be elevated in acute phases vs. remission [35]. Indeed, ELISA data of this study did not demonstrate such significant associations in the different cohorts of APS patients apart from the significant absence of anti-CL IgM in APS patients with pregnancy morbidity. Neither did anti-β2 GPI antibodies detected by both techniques. These data may support the importance of testing for aPL antibodies to pure phospholipids as required by Nash *et al*, demonstrating that omitting the classical anti-CL antibody assay caused 25% of APS patients to be assessed as false-negative [36].

Remarkably, the appearance of aPL IgG antibodies to pure anionic phospholipids detected by MLDA seems to follow a particular pattern in patients with APS. The less frequently found anti-PI IgG antibodies were always accompanied by anti-PS, anti-CL, and anti-β2 GPI IgG. Likewise, anti-PS IgG occurred at significantly higher frequencies together with anti-CL and anti- β2 GPI IgG suggesting a potential epitope spreading of aPL IgG from CL to PS and further PI with the involvement of β2 GPI reactivity. Interestingly, this phenomenon was not consistently observed for aPL IgM. Whether this epitope spreading seen for aPL IgG is confined to the anionic phospholipids employed in the present MLDA only or covers other phospholipid targets like phosphatidylethanolamin remains to be investigated in further studies [37].

## Conclusions

The MLDA technique appears to be an alternative to ELISA for aPL antibody detection in the serological diagnosis of APS. Multiplex detection of aPL antibodies employing membrane surfaces as solid-phase for the antigen immobilization provides the opportunity to develop tests with higher numbers of aPL antibodies improving the still challenging serological diagnosis of APS. IgM antibodies to phospholipids detected by MLDA demonstrate a significant association with cerebrovascular events in APS.

## Abbreviations

APS: antiphospholipid syndrome; aPL: antiphospholipid antibodies; AUC: area under the curve; β2 GPI: beta2-glycoprotein I; CI: confidence interval; CL: cardiolipin; CV: coefficient of variation; DC: disease controls; DVT: deep venous thrombosis; FAS: functional assay sensitivity; LAC: lupus anticoagulant; +LR: positive likelihood ratio; -LR: negative likelihood ratio; mAb: monoclonal antibody; MLDA: multi-line dot assay; NHS: normal healthy subjects; PI: phosphatidylinositol; PS: phosphatidylserine; PVDF: polyvinylidene difluoride; ROC: receiver-operating acharacterisitcs; RT: room temperature; SLE: systemic lupus erythematosus; TIA: cerebral transient ischemic attack.

## Competing interests

Dirk Roggenbuck has a management role and is a shareholder of GA Generic Assays GmbH and Medipan GmbH. Both companies are diagnostic manufacturers. All other authors declare that they have no competing financial interests.

## Authors' contributions

KE, DR, TB, AK, RC, and BL carried out the dot assays. EF, PvL G-RB, and TD conceived of the study, participated in its design and coordination, and helped to draft the manuscript. All authors read and approved the final manuscript.

## Supplementary Material

Additional file 1 **Characterization of the multi-line dot assay in comparison with ELISA**. For evaluation of assay performance, CVs were determined for the four ELISA and the aPL antibody reactivities assessed by MLDA. The anti-CL IgG and IgM ELISAs displayed intra-assay variability ranging from 2.3% to 4.1% and inter-assay variability ranging from 7.4% to 10.3%. The anti- β2 GPI IgG and IgM ELISAs revealed intra-assay variability from 3.3% to 4.5% and inter-assay variability from 5.2% to 6.1%. The intra-assay CVs for a serum reactive with PI, PS, CL, and β2 GPI in the MLDA were 5.2%, 6.8%, 8.3%, and 3.1%, respectively. With respect to ELISA results, the functional assay sensitivity was determined as 3.0 U/ml and 3.5 U/ml for IgG to CL and β2 GPI, respectively, and 2.0 U/ml and 2.5 U/ml for IgM to CL and β2 GPI, respectively. Furthermore, ROC curve analysis revealed the best assay performance for anti-CL IgG antibodies. Click here for file
