# Supplementary material for: Single-step autoantibody profiling in antiphospholipid syndrome using a multi-line dot assay
Source: Arthritis Res Ther. 2011 Jul 21;13(4):R118. doi: 10.1186/ar3421 (PMC3239356; doi:10.1186/ar3421)
Supplement: Additional file 1 — Characterization of the multi-line dot assay in comparison with ELISA. For evaluation of assay performance, CVs were determined for the four ELISA and the aPL antibody reactivities assessed by MLDA. The anti-CL IgG and IgM ELISAs displayed intra-assay variability ranging from 2.3% to 4.1% and inter-assay variability ranging from 7.4% to 10.3%. The anti- β2 GPI IgG and IgM ELISAs revealed intra-assay variability from 3.3% to 4.5% and inter-assay variability from 5.2% to 6.1%. The intra-assay CVs for a serum reactive with PI, PS, CL, and β2 GPI in the MLDA were 5.2%, 6.8%, 8.3%, and 3.1%, respectively. With respect to ELISA results, the functional assay sensitivity was determined as 3.0 U/ml and 3.5 U/ml for IgG to CL and β2 GPI, respectively, and 2.0 U/ml and 2.5 U/ml for IgM to CL and β2 GPI, respectively. Furthermore, ROC curve analysis revealed the best assay performance for anti-CL IgG antibodies. [file ar3421-S1.DOC]

**Additional file**

**Characterisation of the multi-line dot assay**

For evaluation of assay performance, CVs were determined for the 4 ELISA and the aPL antibody reactivities assessed by MLDA. The anti-CL IgG and IgM ELISAs displayed intra-assay variability of 2.3% for a serum with 26.9 U/ml and of 4.1% for a serum with 27.4 U/ml, respectively, and inter-assay variability of 10.3% for a serum with 42.7 U/ml and of 7.4% for a serum with 30.8 U/ml, respectively. The anti- β2GPI IgG and IgM ELISAs revealed intra-assay variability of 4.5% for a serum with 34.3 U/ml and of 3.3% for a serum with 37.9 U/ml, respectively, and inter-assay variabilities of 5.2% for a serum with 41.0 U/ml and of 6.1% for a serum with 23.4 U/ml, respectively. The intra-assay CVs for a serum reactive with PI, PS, CL, and β2GPI in the MLDA were 5.2%, 6.8%, 8.3%, and 3.1%, respectively.

Inter-assay coefficients of variation were measured at different anti-CL and anti-β2GPI IgG and IgM concentrations from 1 to 300 U/ml in 6 different ELISA runs. The functional assay sensitivity representing the lowest antibody concentration with a CV of smaller than 20% was determined as 3.0 U/ml and 3.5 U/ml for IgG to CL and β2GPI, respectively, and 2.0 U/ml and 2.5 U/ml for IgM to CL and β2GPI, respectively.

With respect to ELISA results, ROC curve analysis revealed the best assay performance for anti-CL IgG antibodies, by demonstrating a significant higher area under the curve (AUC) followed by anti-CL IgM, anti-β2GPI IgG, and anti-β2GPI IgM antibodies (Fig. S2).

Table S1: Number of single and multiple aPL antibody positive sera investigating 85 APS patients, 65 control patients, and 79 NHS in ELISA and MLDA

|  |  | Number of positive aPL antibodies in one sample | | | | | | | |
| --- | --- | --- | --- | --- | --- | --- | --- | --- | --- |
|  |  | 1 | 2 | 3 | 4 | 5 | 6 | 7 | 8 |
| ELISA | APS | 9 | 12 | 12 | 17 | - | - | - | - |
|  | DC | 2 | 0 | 0 | 0 | - | - | - | - |
|  | NHS | 5 | 0 | 0 | 0 | - | - | - | - |
| MLDA | APS | 13 | 5 | 20 | 5 | 4 | 9 | 0 | 1 |
|  | DC | 5 | 2 | 0 | 0 | 0 | 0 | 0 | 0 |
|  | NHS | 2 | 5 | 1 | 0 | 0 | 0 | 0 | 0 |

aPL, anti-phospholipid antibody; APS, antiphospholipid syndrome; DC, disease controls; ELISA, enzyme-linked immunosorbent immunoassay; MLDA, multi-line dot assay; NHS, normal healthy subjects

**Figure legends**

**
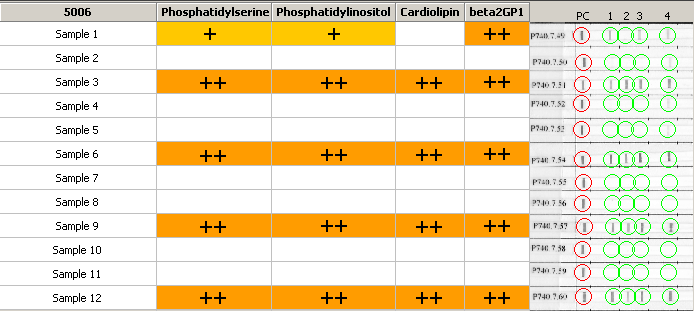
**

**Fig. S1:** Assessment of aPL antibodies to PI, PS, CL, and β_2_GPI by densitometric detection of colored bands generated on PVDF membranes: Serum samples of APS patients (n = 12) were run in the novel MLDA and analysed by the software Dot Blot Analyzer.

**
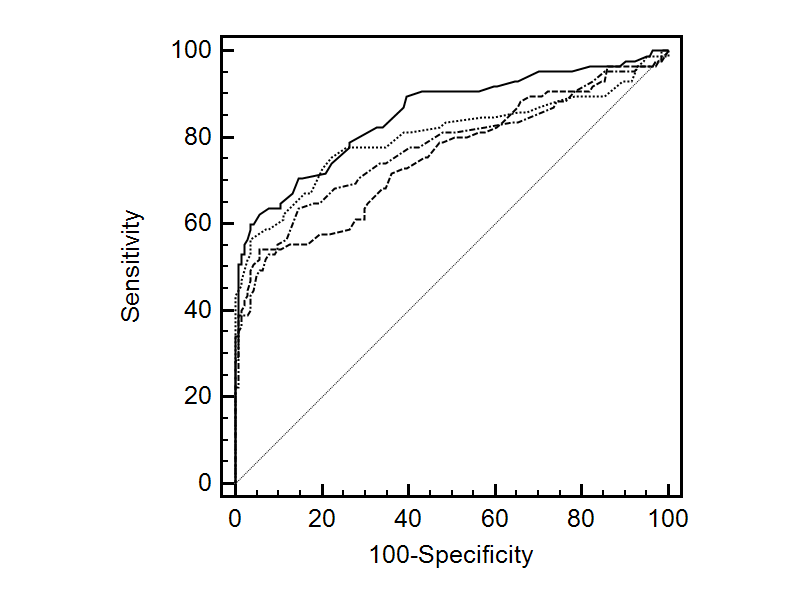
**

**Fig. S2:** Receiver-operating characteristic curve analysis of anti-CL and anti- β_2_GPI IgG and IgM antibodies in 85 APS patients, 65 control patients, and 79 apparently healthy blood donors by ELISA: The AUC for IgG and IgM to CL (bold and dashed lines, respectively) and β_2_GPI (dotted and dotted dashed line, respectively) were 0.853 (95% CI: 0.800 – 0.896), 0.757 (95% CI: 0.696 – 0.811), 0.804 (95% CI: 0.747 – 0.853), and 0.774 (95% CI: 0.715 – 0.827), respectively. The AUC for anti-CL IgG was significantly higher compared with anti-CL IgM, anti-β2GPI IgG, and anti-β2GPI IgG; *P* = 0.001, *P* = 0.050, *P* = 0.010, respectively)
